# Expert review and recommendations on the diagnosis and therapeutic management of eosinophilic granulomatosis with polyangiitis

**DOI:** 10.3389/fimmu.2026.1785849

**Published:** 2026-04-13

**Authors:** Ricardo Blanco, Íñigo Rua-Figueroa, Marina Blanco-Aparicio, Ivette Casafont-Solé, Georgina Espígol-Frigolé, Ismael García-Moguel, Vicente Plaza, Gregorio Soto-Campos

**Affiliations:** 1 Rheumatology, University Hospital Marqués de Valdecilla, Immunopathology group, IDIVAL, Santander, Spain; 2 Rheumatology, University Hospital of Gran Canaria Doctor Negrín, Las Palmas de Gran Canaria, Spain; 3 Pulmonology, University Hospital of A Coruña, La Coruña, Spain; 4 Rheumatology, University Hospital Germans Trias i Pujol, Badalona, Spain; 5 Autoimmune Diseases Department, Hospital Clínic de Barcelona, FCRB-IDIBAPS, Barcelona, Spain; 6 Allergology, University Hospital 12 de Octubre. Instituto de investigación sanitaria i+12, Madrid, Spain; 7 Pulmonology, Hospital de la Santa Creu i Sant Pau, Barcelona, Spain; 8 Pulmonology, University Hospital of Jerez, Jerez de la Frontera, Spain

**Keywords:** biologic therapies, diagnosis, eosinophilic granulomatosis with polyangiitis (EGPA), glucocorticoids, multidisciplinary approach, remission and relapse management, treatment

## Abstract

Eosinophilic granulomatosis with polyangiitis (EGPA) is a rare and heterogeneous immune-mediated disorder in which eosinophilic inflammation, vasculitis, and airway disease coexist in highly variable combinations. In clinical practice, diagnosis remains challenging because no single biomarker reliably captures disease complexity, and therapeutic decisions must still be based on careful integration of clinical, laboratory, imaging, and histopathological findings. In recent years, EGPA management has evolved from a largely glucocorticoid-based approach toward a more phenotype-oriented strategy that distinguishes eosinophilic and vasculitic manifestations and incorporates targeted biologic therapies. This Perspective discusses current challenges and emerging opportunities in EGPA diagnosis and treatment, with emphasis on early recognition, multidisciplinary assessment, glucocorticoid-sparing strategies, and individualized long-term follow-up. Specific recommendations are provided on diagnostic orientation, phenotype-driven therapeutic selection, and the practical use of conventional immunosuppressants and anti–IL-5/IL-5R biologics according to disease severity and organ involvement. In addition, we propose a new treatment algorithm intended to support real-world decision-making and to facilitate a more consistent and structured approach to care. By combining expert recommendations with a forward-looking perspective on evolving therapeutic strategies, this article aims to contribute to safer, more effective, and more personalized management of patients with EGPA.

## Introduction

Eosinophilic granulomatosis with polyangiitis (EGPA), previously known as Churg-Strauss syndrome, is classified as a small-vessel vasculitis associated with anti-neutrophil cytoplasmic antibodies (ANCA) according to the 2012 Chapel Hill consensus classification (1). However, in more than half of the cases, ANCA is absent and the disease may also affect medium-sized vessels. Histologically, EGPA is characterized by eosinophilic, granulomatous, and necrotizing vasculitis (2).

EGPA is a rare condition, with a global annual incidence of approximately 1.7 cases per million people, and a prevalence of 15.6 per million (3, 4). The clinical presentation is highly variable, with respiratory tract involvement being the most common manifestation. Patients almost always have a history of bronchial asthma and often have also a chronic rhinosinusitis with nasal polyps (CRSwNP) (5). While these manifestations may not always reflect the activity of the vasculitis itself, they are considered part of the clinical picture (5, 6). The heterogeneous nature of EGPA poses a significant challenge in both diagnosis and treatment, with the disease frequently evolving through different clinical stages that require different management strategies.

The disease typically evolves through three phases: prodromic, eosinophilic, and vasculitis. (1) The prodromic phase often includes late-onset asthma and allergic rhinosinusitis, which may precede the onset of vasculitis by up to 9 years (7). (2) The eosinophilic phase is characterized by eosinophilic infiltration into various organs, particularly the lungs, which are affected in up to 70% of patients (7). Gastrointestinal involvement with eosinophilic infiltrates can lead to nonspecific symptoms such as abdominal pain, diarrhea, and gastrointestinal bleeding. Cardiac involvement is also common, with eosinophilic infiltration causing myocarditis, pericarditis, valve disease, and conduction abnormalities (7). Notably, patients with predominant eosinophilic involvement often test negative for ANCA (7). (3) The vasculitis manifestations include generalized symptoms such as fatigue and fever, alongside more specific vasculitis signs, including peripheral neuropathy (mononeuritis multiplex), skin manifestations (palpable purpura, nodules or ulcerations), and renal involvement, which, while less frequent, can be severe (7).

**Figure 1:**
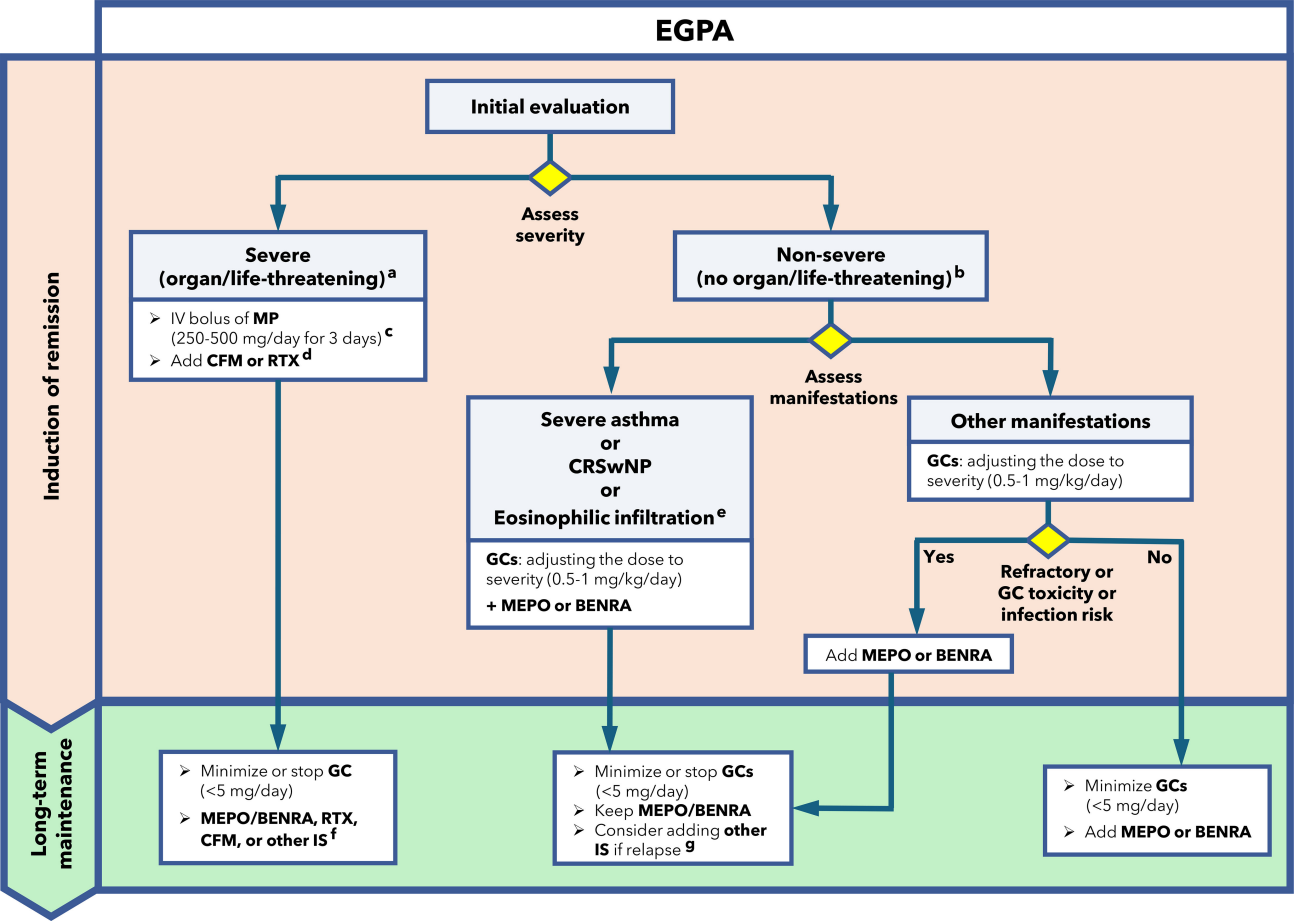
Proposed treatment algorithm. (**a)**: Renal involvement (glomerulonephritis), central nervous system involvement, mononeuritis multiplex, cardiac involvement, retro-orbital disease, mesenteric ischemia, gastrointestinal bleeding, limb ischemia, alveolar hemorrhage (70). **(b)**: Rhinosinusitis, asthma, uncomplicated cutaneous disease, mild inflammatory arthritis, myositis limited to skeletal muscle, episcleritis, non-cavitating pulmonary nodules (70). **(c)**: At clinician’s criteria (1000 mg/day dose may be exceptionally used for most severe cases). **(d)**: Prioritize RTX over CFM in patients with reproductive potential, or when prior CFM exposure has reached an individual cumulative dose associated with higher toxicity/complication risk (22). **(e)**: The term “eosinophilic infiltration” refers to predominantly eosinophilic organ involvement (e.g., lung, heart, gastrointestinal tract) without overt vasculitis. **(f)**: Anti–IL-5/IL-5R biologics, AZA, methotrexate, mycophenolate mofetil, or RTX may be considered on a case-by-case basis (22). **(g)**: AZA, methotrexate, mycophenolate mofetil, or RTX may be considered on a case-by-case basis (22). AZA: azathioprine; BENRA: benralizumab; CFM: cyclophosphamide; CRSwNP: chronic rhinosinusitis with nasal polyps; EGPA: eosinophilic granulomatosis with polyangiitis; GCs: glucocorticoids; IS: immunosuppressant; IV: intravenous; MP: methylprednisolone; MEPO: mepolizumab; RTX: rituximab.

In this Perspective, we convened a multidisciplinary expert panel and combined a targeted literature synthesis with a structured consensus process to generate practical, phenotype-oriented recommendations. We focused on diagnostic red flags, phenotype differentiation, glucocorticoid-sparing strategies, and the role of biologics in induction and maintenance. Full details of the methodology, such as panel composition, search strategy, eligibility criteria, and consensus procedures, are provided in the **Supplementary Material**. The results of this work include a set of consensus recommendations (**Table 1**) and a pragmatic treatment algorithm (**Figure 1**) aimed at improving real-world EGPA care.

**Table 1:** Summary of expert recommendations.

Recommendation	Description
1. Early diagnosis based on clinical suspicion	Prompt diagnosis of EGPA is decisive, in order to start early treatment, preventing organ damage. Awareness of red flags (9), especially in the context of asthma and sinusitis, which are common early symptoms, is crucial.
2. Early involvement of a multidisciplinary team	Create specialized multidisciplinary clinics for EGPA patients, involving pulmonologists, rheumatologists, cardiologists, neurologists, and immunologists.
3. Personalized treatment plans based on disease phenotype	Develop individualized treatment strategies that are tailored to the clinical phenotype (vasculitic or eosinophilic), severity, comorbidities, and response to therapy.
4. Use high-dose GCs for induction in severe cases	Initiate induction therapy with high doses of GCs in severe cases, such as those with myocardial or nervous system involvement.
5. Minimize long-term GC use	The goal is to minimize the cumulative dose of GCs to reduce side effects by early introduction of GC-sparing agents.
6. Employ biologics like anti−IL−5/IL−5R for eosinophilic phenotypes	Use anti−IL−5/IL−5R agents like mepolizumab or benralizumab for patients with predominant eosinophilic features to reduce eosinophil counts and control disease activity. These agents can be useful also for remission maintenance phase for patients with severe, even vasculitis phenotype
7. Regular monitoring for comorbidities	Screen for comorbidities such as previous or ongoing infections, cardiovascular disease, and osteoporosis regularly, especially in patients needing prolonged GC therapy.
8. Prevention of infections and vaccination	Implement preventive measures for infections, including vaccination and screening for latent infections in patients receiving immunosuppressive therapies.
9. Use a combination therapy approach for refractory cases	For patients who do not respond to monotherapy, consider combining biologics with conventional immunosuppressive agents pursuing a synergistic effect.
10. Regular follow-up with a coordinated care plan	Ensure close follow-up through a coordinated, multidisciplinary care team to track disease progression, prevent relapses, and adjust therapy as needed.

EGPA: eosinophilic granulomatosis with polyangiitis; GCs: glucocorticoids.

## Diagnosis and classification

### EGPA diagnosis

We recommend a pragmatic, stepwise approach to diagnosis that acknowledges the disease’s heterogeneity and the absence of standardized, validated diagnostic criteria (8). In practice, we consider EGPA in any patient (aged ≥6 years) with otherwise unexplained eosinophilia, particularly when absolute eosinophil counts exceed 1,000 cells/µL in the absence of treatment or 500 cells/µL if already exposed to medications that may lower eosinophils (e.g., GCs) (9). The co-occurrence of asthma and/or nasal polyposis should heighten suspicion above other eosinophilic disorders (**Table 1**).

Once EGPA is suspected, we ground the diagnosis in a composite assessment of clinical, laboratory, imaging, and histopathological data. Clinically, we prioritize features such as nasal involvement (CRSwNP), airway disease (asthma), and neurologic involvement (mononeuritis multiplex). Laboratory criteria center on eosinophilia, either in peripheral blood or as tissue-predominant infiltrates on biopsy. Although no single biomarker is definitive, eosinophilia remains the most informative signal—particularly when counts surpass 1,500 cells/µL, namely, hypereosinophilia (10). Ancillary serum markers (e.g., IgE, IgG4, CRP) may support the evaluation but lack specificity. The absence of ANCA does not exclude EGPA; therefore, a comprehensive, expert-led synthesis of clinical, radiographic, and histopathological findings is essential.

We also emphasize rigorous differential diagnosis to systematically exclude alternative causes of hypereosinophilia (5, 7, 10–12): other eosinophilic syndromes (notably hypereosinophilic syndrome (HES); pulmonary conditions such as allergic bronchopulmonary aspergillosis and idiopathic eosinophilic pneumonia; drug hypersensitivity; paraneoplastic processes; and parasitic infections. In clinical practice, differentiating ANCA-negative EGPA from idiopathic HES may be particularly challenging, especially when eosinophilic organ involvement predominates. Histologically, EGPA is defined by necrotizing vasculitis and extravascular granulomas; however, biopsy confirmation is not always feasible because affected tissue may be difficult to access and diagnostic yield can be limited. In this context, the presence of asthma or CRSwNP strongly supports EGPA, whereas these features are uncommon in idiopathic HES. Although both conditions may present with organ- or life-threatening disease, certain patterns may help orientation: cardiac involvement in idiopathic HES is classically driven by eosinophil-mediated toxicity, with a propensity for mural thrombosis, systemic embolism, and progression to endomyocardial fibrosis, and advanced disease may manifest as restrictive cardiomyopathy. Neurological involvement in EGPA typically reflects peripheral nerve disease (e.g., mononeuritis multiplex or polyneuropathy), whereas thromboembolic neurological complications (including ischemic stroke and systemic embolism) are reported more often in HES. When a clear distinction remains uncertain after comprehensive evaluation, application of the ACR/EULAR classification criteria for EGPA may be used to support diagnostic orientation (6). Distinction from the other ANCA-associated vasculitis (granulomatosis with polyangiitis and microscopic polyangiitis) and from IgG4-related disease is equally important (12).

### Clinical phenotype differentiation

Several classification criteria have been developed for ANCA-associated vasculitis: American College of Rheumatology (ACR) in 1990 (13), the European Medicines Agency (EMA) in 2007 (14), and the ACR/European Alliance of Associations for Rheumatology (EULAR) criteria in 2022 (6). The most recent criteria have demonstrated a sensitivity of 75% and a specificity of 99% for EGPA classification, and it is important to note that MPO-ANCA positivity is not part of these criteria (6), since no clear phenotypic association can be demonstrated (15).

EGPA can be categorized into different clinical phenotypes based on the predominant manifestations and biomarkers. One of the current challenges of EGPA is the identification and differentiation of these clinical phenotypes. Eosinophilia is the most important biomarker for lung and extrapulmonary disease classification and prognosis (16). The eosinophilic phenotype, which is frequently ANCA-negative, involves eosinophilic infiltration primarily affecting the lungs (a lung-dominant EGPA has been described (17)), gastrointestinal tract and heart (5, 10).

The vasculitis phenotype, usually associated with ANCA positivity, frequently presents with more severe disease with systemic symptoms, including fever, myalgias, and elevated acute-phase reactants, with predominant cutaneous, renal, and peripheral neuropathy involvement (10). Not infrequently, overlap between phenotypes often occurs, complicating clinical interpretation and treatment decisions (5, 11, 12).

## EGPA therapeutic management

### Treatment objectives

Therapeutic goals should be centered on suppressing active inflammation, preserving organ function, and minimizing treatment-related harm. Remission can be induced by systemic GCs—alone or combined with conventional IS—yet substantial relapse rates have been reported, and inadequate symptom control with accruing organ damage is frequently observed (3, 18, 19). Control of vasculitic manifestations is often achieved with traditional regimens, whereas upper-airway ear, nose and throat (ENT) disease and asthma are commonly undertreated.

Complete remission should be understood as absence of clinical activity with normalization of laboratory parameters and, ideally, without ongoing GC or immunosuppressants. In practice, “drug-free” remission is uncommon (≈15–30% in published series) (20, 21). Consistent with current guidelines, minimal maintenance therapy may be required; low-dose prednisone (up to 5 mg/day, or equivalent) is considered acceptable when needed to control vasculitis manifestations (22).

Persistent uncertainties in defining treatment targets are recognized. Existing remission constructs have been derived predominantly from systemic/vasculitic domains (6, 8, 13, 14). No consensus has been reached on whether asthma or ENT symptoms should be included, despite their tendency to persist or flare independently of systemic disease activity. Whether any GC use should be allowed within a remission definition also remains debated.

For operational clarity, relapse should be defined as either active vasculitis with Birmingham Vasculitis Activity Score (BVAS) > 0 or active asthma or ENT disease that prompts an increase in prednisone dose, initiation/escalation of immunosuppressive therapy, or hospitalization (6, 8, 13, 14, 23). Asthma and upper-airway control may fluctuate for reasons unrelated to vasculitis; therefore, isolated worsening of asthma or sinusitis and/or rises in blood eosinophils that do not necessitate those actions should not be classified as relapse, although targeted management is often appropriate given their clinical impact.

### Conventional treatment with GCs and immunosuppressive agents

Systemic GCs remain the cornerstone of remission induction in EGPA (18, 24). Current practice favors initiating high-dose GCs at 0.5–1 mg/kg/day (maximum 80 mg/day), with a progressive tapering over 3–6 months toward withdrawal or maintenance <5 mg/day, adjusted to severity and response (18, 24). In severe presentations, such as myocardial involvement, diffuse alveolar hemorrhage, or neurologic disease, intravenous pulses of methylprednisolone are recommended as part of induction (18, 24). Despite widespread use, a standardized, evidence-based regimen for tapering and duration has not been established (18, 24). Observational data indicate that unsuccessful GC discontinuation is often driven by suboptimal asthma control, underscoring the need to optimize airway disease specific management; moreover, GCs may be insufficient to control asthma or eosinophilic rhinosinusitis in a subset of patients (25, 26).

In line with GC management, the guiding principle is to use the lowest effective dose for the shortest period compatible with disease control (18, 24). Nevertheless, long-term GC exposure remains common (reported in up to 85% of patients) and does not invariably prevent relapses (18, 27). Prolonged GC therapy carries substantial risk, including serious infection, osteoporotic fractures, hypertension, diabetes, adrenal insufficiency, glaucoma and cataracts (28–30). Early introduction of GC-sparing agents, close monitoring, and structured patient education to avoid unsupervised GC use are therefore advised (18, 24, 31).

When disease is severe or refractory to GCs or GC-dependent, immunosuppressive induction is commonly employed. Cyclophosphamide (CFM) remains a key option to rapidly control systemic vasculitis inflammation and reduce GC burden, particularly with major organ involvement (18, 24, 32). However, its use requires careful risk–benefit assessment given associations with increased malignancy risk, serious infection and infertility (33–35). These considerations support individualized strategies that integrate clinical phenotype, disease severity, comorbidities, and patient preferences to balance efficacy with long-term safety. Despite declining use, conventional lower-toxicity immunosuppressants, such as methotrexate, and azathioprine, remain appropriate options for selected patients (36, 37).

For patients requiring prolonged GCs or immunosuppressants treatment, preventive care is essential: ensure up-to-date immunizations, screen for latent infections, and implement osteoporosis, diabetes, and cardiovascular risk mitigation strategies (38).

### Biologic therapies and targeted approaches

Recent advancements in the treatment of EGPA have focused on targeting specific cytokines and/or immune cells involved in the pathogenesis of EGPA. Over the past decade, biologic therapies have represented a significant shift in the management of EGPA, providing targeted treatments that reduce dependence on GCs and conventional immunosuppressants.

B-cell depletion therapies, such as rituximab, a monoclonal antibody targeting CD20-positive B-cells, have been used with success, particularly in patients with ANCA-positive EGPA (39). Rituximab provides an alternative to reduce CFM-related toxicity for EGPA patients, but it frequently requires keeping low GCs dosing to maintain disease control (40) and it is associated with potential complications, including infection risk (41, 42). Nevertheless, according to a recent randomized controlled trial, rituximab was not inferior to conventional immunosuppression for remission induction in EGPA patients, showing similar safety profile (43).

An alternative and most promising area of intervention involves targeting interleukin-5 (IL-5) or its receptor (IL-5R). IL-5 is a cytokine that plays a pivotal role in the differentiation, survival and activation of eosinophils (44). This approach, focusing on cytokine inhibition, provides an important alternative to traditional treatments and paves the way for more personalized and safer therapies, based on patient-specific characteristics. Modulating eosinophilic- mediated inflammation has become a central strategy in EGPA, with two different approach available: mepolizumab which binds and neutralizes IL-5, reducing eosinophil maturation and survival (45), whereas benralizumab targets IL-5Rα on eosinophils and triggers antibody-dependent cell-mediated cytotoxicity, leading to near-complete eosinophil depletion (46).

Mepolizumab and benralizumab display broadly similar favorable safety profiles (47–51), and both approaches have been associated with improved long-term disease control, fewer relapses, and meaningful GC-sparing, both in non-severe patients and in those with a history of severe disease (50–57). Nevertheless, while real-world studies indicate broadly similar efficacy between the two biologics (58–60), direct comparisons showed advantages for benralizumab in GC withdrawal, including patients with EGPA and severe asthma (61, 62). Case-based reports also describe successful responses to benralizumab after inadequate control with mepolizumab (63, 64), and benralizumab benefit in difficult domains, such as neuropathy and eosinophilic myocarditis (64–66). On the other hand, the efficacy of benralizumab in CRSwNP may be less substantiated due to certain inconsistencies in the results from the clinical trials up to date (67–69). However, no differences between mepolizumab and benralizumab were observed in terms of SNOT-22 in the comparative assay MANDARA (61).

### Recommended treatment strategy

We propose the treatment algorithm displayed in **Figure 1**. The main goal of EGPA management is to achieve and maintain remission with the least amount of treatment-related toxicity, avoiding organ damage and improving quality of life. The treatment regimen should be tailored to disease severity, clinical manifestations, the predominant phenotype, comorbidities, risk profile, and individual response to induction therapy.

For patients with severe EGPA, defined as organ- or life-threatening disease (70) (e.g., glomerulonephritis, mononeuritis multiplex, alveolar hemorrhage, severe cardiac involvement, central nervous system involvement), we recommend treating with high-dose intravenous methylprednisolone pulses of 250–500 mg/day (or up to 1000 mg/day in the most critical cases) for 3 days, combined with immunosuppressive agents, such as CFM or rituximab. Rituximab is favored in relapsing disease, ANCA positive, in patients with reproductive potential, or when prior CFM exposure approaches a dose-dependent cumulative threshold of concern (71), while CFM remains appropriate when rapid effect is prioritized or rituximab is contraindicated (22). In eosinophilic myocarditis without clear vasculitis features, anti−IL−5/IL−5R biologics may be added even without immunosuppressants.

Treatment for patients with non-severe EGPA (non–organ-threatening presentations) generally begins with GC induction, adjusting the dose to severity (0.5–1 mg/kg/day). In refractory cases, or when GC toxicity or infection risk is present, we recommend adding anti−IL−5/IL−5R biologics. In patients with severe asthma, CRSwNP, or eosinophilic infiltration, we recommend starting GC induction in combination with anti−IL−5/IL−5R biologics, since they significantly improve outcomes, provide greater control over eosinophilic inflammation, and reduce the need for systemic GCs.

For long-term management, we recommend keeping IL-5/R biologics in all cases (**Figure 1**), while GC treatment should be reduced to the lowest possible dose (<5 mg/day) or completely withdrawn. Maintenance treatment should be individualized according to phenotype, organ involvement, ANCA status, comorbidities, prior induction regimen, and relapse risk. In severe cases, RTX should be considered preferentially for patients with a predominantly vasculitis phenotype (particularly those with MPO-ANCA positivity, renal involvement, or a relapsing vasculitis course) and it may be especially appropriate when RTX has already been used successfully for induction. Conversely, in patients with ANCA-negative disease and predominantly eosinophilic manifestations (e.g., cardiac involvement without clear vasculitis features), maintenance may be appropriately achieved with GC-sparing strategies such as anti–IL-5/IL-5R biologics or conventional immunosuppressants (e.g., AZA, MTX, MMF), based on clinical context and tolerability.

In any case, preventing and managing relapses is another critical aspect of EGPA care. Early recognition of relapses, such as eosinophilic exacerbations or vasculitis flares, is crucial for effective intervention. If a relapse occurs, step-up therapy (including additional immunosuppressives or biologics) may be needed. In cases with extrapulmonary organ involvement (e.g., myocarditis), an immunosuppressant can be added depending on severity. Close follow-up is recommended to monitor eosinophil counts, asthma symptoms, and signs of systemic activity, allowing for timely intervention and adjustment of therapy.

## Multidisciplinary approach for diagnosis and follow-up

Given EGPA’s complexity and the diversity of its manifestations, a multidisciplinary model is essential (**Table 1**). We advocate anchoring care in a core team of pulmonology, rheumatology, internal medicine and allergy/clinical immunology experts responsible for early diagnosis, phenotypic and severity stratification, and longitudinal coordination. Depending on the patient’s phenotype and organ involvement, additional specialties should join the team: ENT for CRSwNP; dermatology for cutaneous vasculitis and eosinophilic dermatoses; cardiology for myocardial/pericardial disease; and neurology for peripheral or central nervous system involvement.

A thorough review of biopsies by pathology experts is crucial given the difficulty in demonstrating vasculitic pathology, as well as the involvement of hospital pharmacy to facilitate the timely availability of targeted therapies. In some cases, collaboration with hematology departments may also be required, particularly when it comes to ruling out hypereosinophilic syndrome and other hematological differential diagnoses. We recommend establishing specialized EGPA clinics in reference centers and structured coordination pathways between respiratory and services involved in systemic disease care, with streamlined referral to organ-specific specialists as needed. This collaborative framework ensures that each manifestation is addressed promptly and that individualized treatment plans are consistently implemented and reviewed.

In patients with persistent diagnostic uncertainty between ANCA-negative EGPA and idiopathic HES after comprehensive evaluation and exclusion of secondary causes of hypereosinophilia, management should be individualized and periodically reassessed as additional clinical, laboratory, imaging, and (when available) histopathological data become available. Because both conditions may present with cardiac, pulmonary, and neurologic involvement and may show benefit with eosinophil-targeted therapy, anti–IL-5/IL-5R biologics may be considered as a pragmatic steroid-sparing option when eosinophilic inflammation predominates, while recognizing that vasculitis-directed immunosuppressive strategies (e.g., CFM or rituximab) are not routinely used for idiopathic HES and should be reserved for scenarios with compelling evidence or a high clinical likelihood of vasculitis and/or organ- or life-threatening disease. In these situations, the balance of expected benefit and competing risks should be weighed in a multidisciplinary setting, preferably in expert centers.

## Discussion

Despite advancements in treatment, several challenges persist in the management of EGPA. The diagnosis remains complex and mainly relying on clinical expert judgment. While clinical criteria, together with laboratory and imaging studies, have improved diagnostic accuracy, there is still a need for further research to identify reliable biomarkers that can assist in diagnosing and monitoring disease progression. Additionally, the heterogeneity of EGPA, with its diverse clinical phenotypes, poses a significant challenge in providing a standardized diagnostic approach.

In this regard, increasing attention is being paid to the concept of EGPA as a disease spectrum composed of partially distinct immunopathogenic endotypes rather than a single entity. In particular, genetic studies suggest that ANCA-positive and ANCA-negative EGPA may differ not only clinically, but also biologically, with ANCA-positive disease sharing features with other autoimmune ANCA-associated vasculitis, whereas ANCA-negative disease appears to be more closely linked to eosinophilic and type 2 inflammatory mechanisms (6, 72). Similarly, cluster analyses based on clinical and serological features support a more nuanced stratification of EGPA beyond a simple ANCA-based dichotomy (15). From a precision medicine standpoint, current genome wide association data already support the existence of partly distinct ANCA positive and ANCA negative EGPA endotypes, as exemplified by the work of Lyons et al. (72). Beyond single loci, future integration of common and rare genetic variants, copy number variation, DNA methylation signatures and polygenic risk scores may help refine endotype classification, improve prognostic stratification and ultimately inform treatment selection. At present, however, these genomic tools remain exploratory and are not ready for routine clinical use, underscoring the need for validation in larger, longitudinal EGPA cohorts.

This evolving view has important implications for biomarker development. Although blood eosinophilia remains the most useful biomarker in routine practice, its specificity is limited and it does not fully capture disease heterogeneity. Emerging studies from proteomics, tissue immune profiling, and translational immunology are beginning to identify candidate markers that may refine diagnosis and endotype classification. Proteomic analyses have suggested potential serum signatures able to distinguish EGPA from severe asthma, while tissue-based studies indicate that pulmonary EGPA may be characterized by a combined type 2 and immunoregulatory immune program, including eosinophilic inflammation, IgG4-rich plasma cells, and macrophage-associated pathways (73, 74). Likewise, exploratory autoantibody studies suggest that biomarkers beyond MPO-ANCA may help define biologically distinct subsets, although none are ready for routine clinical use (75). Importantly, several candidate biomarkers—including eotaxin-3, TARC/CCL17, IgG4, and broader cytokine/chemokine panels—have shown limited ability to consistently discriminate active from inactive disease, especially in patients receiving glucocorticoids, underscoring the need for further validation (76, 77).

The management of EGPA has undergone a significant transformation with the introduction of biologic therapies, offering patients a more targeted and effective and safer approach (78). The emergence of anti-IL5/5R biologic agents, like mepolizumab and benralizumab, has changed the therapeutic landscape by targeting specific aspects of the disease, such as eosinophil-mediated inflammation. These safe and effective agents not only reduce the need for systemic GCs, but also improve clinical outcomes, including reducing exacerbations and preventing relapses (62). Their introduction marks a significant step toward a more individualized approach to treating EGPA, with the potential to reduce long-term complications associated with traditional immunosuppressive therapies.

The treatment of EGPA is increasingly shifting towards more personalized and targeted approaches, with combination therapies poised to play a crucial role. This is particularly true for patients with severe disease or mixed phenotypes or those who fail to respond adequately to monotherapy. Combining biologic agents, such as mepolizumab or benralizumab, with immunosuppressive drugs like methotrexate or azathioprine, could potentially offer a synergistic effect, addressing both eosinophilic and vasculitis manifestations of the disease. Early trials of combination therapies have shown promising results, suggesting better disease control and reduced relapse rates (62).

In the future, combination therapies could also address both the eosinophilic and vasculitis components of EGPA. For example, combining rituximab with anti−IL−5/IL−5R biologics may help control both eosinophilic inflammation and vasculitis processes, improving outcomes for patients with more complex disease presentations (79). Furthermore, the role of the microbiome and gut health in autoimmune diseases could offer new therapeutic avenues, particularly for patients with significant gastrointestinal involvement or those who have not responded to conventional treatments (80). Early work specifically addressing the gut microbiota and associated mucosal immune responses in EGPA suggests that disease associated dysbiosis may contribute to immune dysregulation in this setting (81), although it remains unclear whether these changes are causal or secondary and whether microbiota targeted interventions could meaningfully modify disease course.

Despite the effectiveness of anti−IL−5/IL−5R biologics, ongoing research is crucial to expand our understanding of EGPA and refine treatment strategies. While much of the current focus has been on targeting eosinophilic inflammation, it is becoming clear that other immune cells, soluble mediators and pathways also contribute to the disease. Upstream Thymic Stromal Lymphopoietin is an alarm signal in the airway important in causing vasculitis and its blockade is being tested with tezepelumab in a phase II, randomized, double-blind study in EGPA (RACEMATE; NCT06230354), which evaluates remission using BVAS-based criteria with constrained prednisone dosing (82). In parallel, JAK-STAT pathway modulation is under investigation in a phase II randomized trial of the selective JAK1 inhibitor NS-229 in EGPA (NCT06046222), reflecting interest in targeting broader immune signaling beyond the IL-5 axis (83).

Future therapies may target Th2 cytokines, such as IL-4 and IL-13, which are integral to the disease’s pathophysiology (84). Additionally, emerging research into T-cell modulation and IL-17 inhibition may provide new avenues for treatment, especially in patients with a mixed phenotype (85). Ultimately, the integration of genomics, transcriptomics, proteomics, serological markers, and longitudinal clinical phenotyping may allow the development of systems-level models capable of improving diagnostic accuracy, refining endotype-driven treatment selection, and anticipating relapse or treatment response. Although this precision medicine approach is still in development, it represents one of the most promising directions for future research and may ultimately reshape the diagnosis and therapeutic management of EGPA (15, 72, 73).

## Conclusion

In summary, while much progress has been made in the treatment of EGPA, there is still significant knowledge to be gained. The advent of biologic therapies has opened new therapeutic horizons, but challenges remain in optimizing treatment strategies, refining diagnostic criteria, and addressing the diverse phenotypic manifestations of the disease. Early identification and proper managing of EGPA manifestations (ideally within a multidisciplinary framework) may improve patient outcomes, enhance disease control and reduce relapse frequency. A coordinated approach involving specialized consultations and clear referral pathways is recommended to optimize patient outcomes. Future research will be critical in advancing our understanding and improving the management of EGPA.

## Data Availability

The original contributions presented in the study are included in the article/**Supplementary Material**. Further inquiries can be directed to the corresponding author.
