# Supplementary material for: Expert review and recommendations on the diagnosis and therapeutic management of eosinophilic granulomatosis with polyangiitis
Source: Front Immunol. 2026 Apr 13;17:1785849. doi: 10.3389/fimmu.2026.1785849 (PMC13111300; doi:10.3389/fimmu.2026.1785849)
Supplement: Supplementary file 1 [file DataSheet1.docx]

**SUPPLEMETARY MATERIAL**

**Expert review and recommendations on the diagnosis and therapeutic management of eosinophilic granulomatosis with polyangiitis**

**Methods**

## **Expert panel**

A multidisciplinary panel of eight experts included three specialists in rheumatology, three pulmonologists, one allergy expert, and one internal medicine specialist. All these experts had extensive clinical experience and had contributed significantly to EGPA research, especially regarding diagnosis and treatment approaches.

## **Literature review and data collection**

The literature review was conducted by searching for major scientific databases, including PubMed, Scopus, Google Scholar, and Semantic Scholar, for studies published in the last 10 years. Key thematic areas were covered in the search, including clinical manifestations, diagnostic criteria, treatment strategies, and emerging therapies for EGPA. The inclusion criteria for the literature review encompassed clinical trials, systematic reviews, meta-analyses, and observational studies published in English. In addition, national reports and strategic plans were incorporated to ensure a comprehensive understanding of the disease's management. Studies not focused on EGPA or lacking relevant clinical data, according to experts’ opinion, were excluded.

The search strategy was predefined around five thematic blocks—Introduction, Oral Glucocorticoids, Immunosuppressive Therapies, Anti–IL-5/IL-5R Therapies, and Other Treatments—and limited to studies published in the past 10 years. For each block we combined disease terms (“Eosinophilic Granulomatosis with Polyangiitis,” “EGPA,” “allergic granulomatosis,” “Churg-Strauss syndrome”) with intervention or management terms, applying a 10-year recency filter and adapting Boolean strings to each database. For the Introduction block, we used queries such as: (("Eosinophilic Granulomatosis with Polyangiitis"[MeSH] OR "EGPA" OR "allergic granulomatosis" OR "Churg-Strauss syndrome") AND ("therapeutics" OR "treatments" OR "therapy" OR "treatment") AND (y_10[Filter])). For Oral Glucocorticoids, we added corticosteroid terms—("oral glucocorticoids" OR "oral corticosteroids" OR "OCS")—to the core EGPA string with the same 10-year filter. For Immunosuppressive Therapies, we combined the EGPA core with ("immunosuppressors" OR "methotrexate" OR "azathioprine" OR "cyclophosphamide") and, separately, with "rituximab," each constrained to the last decade. For anti–IL-5/IL-5R Therapies, we paired the EGPA core with ("anti-IL-5" OR "anti-IL-5R") and conducted focused searches on "mepolizumab" and "benralizumab," again limited to the last 10 years. Finally, we ran a broader “Other Treatments” block to capture additional or emerging interventions. To ensure comprehensive coverage, we supplemented database outputs with targeted citation chasing and select grey literature (e.g., national reports and strategic plans) relevant to diagnosis, care pathways, and access considerations

## **Consensus process**

In addition to the literature review, the data collection process involved expert input. The experts discussed a series of questions covering several critical areas, such as the identification of useful biomarkers and diagnostic tools, the evaluation of various treatment strategies, and the assessment of clinical phenotypes and their influence on treatment decisions. Experts also discussed therapeutic goals, including remission induction, relapse prevention, and strategies for GC reduction or discontinuation. Furthermore, the role of a multidisciplinary approach in managing EGPA was explored, along with recommendations for improving patient care.

The primary objective was to create a practical and applicable set of comprehensive recommendations for healthcare providers managing EGPA patients, focusing on clinical feasibility and relevance. The panel reviewed the most recent evidence, shared insights from their clinical experiences, and debated key aspects of EGPA management until reaching consensus on the clinical recommendations for the manuscript. No formal voting or ranking methods were used. Instead, the experts engaged in detailed discussions, resolving disagreements through collaborative debate and consensus-building.
